# HIF-1α Signaling in Uterine Fibroids: A Central Integrator of Hypoxic, Hormonal, and Fibrotic Pathways

**DOI:** 10.3390/oxygen6020009

**Published:** 2026-04-17

**Authors:** Sruthi Tatavarthi, Valentina Vanos, Abigail Lepsch Combs, Alvina Pan, Mahita Saini, Mostafa A. Borahay

**Affiliations:** 1 Johns Hopkins University Krieger School of Arts and Sciences, Baltimore, MD 21218, USA; 2 Department of Population, Family, and Reproductive Health, Bloomberg School of Public Health, Baltimore, MD 21205 USA; 3 Department of Gynecology and Obstetrics, Johns Hopkins University School of Medicine, Baltimore, MD 21205, USA; 4 Johns Hopkins University Advanced Academic Programs, Baltimore, MD 21218, USA

**Keywords:** HIF-1α, uterine fibroids, VEGF, mTOR, TGF-β/SMAD pathway, reactive oxygen species, GLUT1

## Abstract

Uterine fibroids (leiomyomas) are common benign smooth muscle tumors that impose substantial symptom burden and healthcare costs worldwide. Although uterine fibroid (leiomyoma) pathogenesis is multifactorial, hypoxia has emerged as a key feature of the uterine fibroid (leiomyoma) microenvironment, particularly within poorly perfused tumor cores. Hypoxia-inducible factor-1α (HIF-1α) is a central transcriptional regulator of cellular adaptation to low oxygen and coordinates downstream programs that support angiogenesis, metabolic reprogramming, cell survival, and extracellular matrix (ECM) remodeling. In uterine fibroids (leiomyomas), these HIF-1α–dependent processes intersect with steroid hormone signaling, growth factor pathways, inflammatory mediators, and redox imbalance, together promoting tumor persistence and progressive fibrosis. This review synthesizes the molecular regulation of HIF-1α, highlights major HIF-linked effector pathways relevant to uterine fibroid (leiomyoma) biology, and emphasizes mechanistic crosstalk with estrogen- and progesterone-responsive signaling, TGF-β/SMAD-driven fibrosis, NF-κB-mediated inflammation, and metabolic checkpoint pathways including mTOR and AMPK. Finally, we evaluate emerging therapeutic strategies that target HIF-1α directly or indirectly through upstream regulators.

## 1. Introduction

Uterine fibroids, also known as leiomyomas, represent a substantial health concern for the majority of reproductive age women worldwide [1]. These benign tumors, originating from the myometrium, contribute to a range of distressing symptoms while posing considerable challenges to healthcare systems due to diagnosis, treatment, and management-based costs [2]. While the precise etiology of uterine fibroids (leiomyomas) remain elusive, a combination of genetic predisposition, hormonal influences, growth factors, and environmental factors are believed to play critical roles in their development and progression.

As uterine fibroids (leiomyomas) undergo rapid growth, cellular proliferation can exceed the capacity of the existing blood vessels needed to maintain the tissue, which creates regions of the tissue that have low oxygen supply. Among these contributors, hypoxia (reduced oxygen tension) has emerged as a recurring and biologically plausible feature of uterine fibroid (leiomyoma) biology, particularly as uterine fibroids (leiomyomas) enlarge and outpace their blood supply [3,4]. Structural and mechanical constraints, including abnormal vascular architecture and large-scale fibrosis, compress vessels and limits oxygen diffusion. A hypoxic microenvironment can promote persistent growth and remodeling by activating hypoxia-inducible factor-1α (HIF-1α), a transcriptional regulator that coordinates angiogenic, metabolic, survival, and extracellular matrix programs [5]. While HIF-1α signaling has been extensively characterized in cancer and fibrotic disease, its role in uterine fibroids (leiomyomas), especially its integration with hormonal, inflammatory, and metabolic signaling, remains less clearly defined. This review aims to provide a comprehensive overview of the involvement of HIF-1α in uterine fibroids (leiomyomas) by streamlining the signaling cascade and exploring its relationship with other hormonal, inflammatory, and metabolic key signaling pathways, to identify potential therapeutic targets for uterine fibroid (leiomyoma) management.

## 2. Hypoxia-inducible factor-1α

Hypoxia-inducible factor 1 alpha (HIF-1α) is a pivotal transcription regulator that mediates cellular adaptation and programming to reduced oxygen availability [5–7]. HIF-1 functions as a heterodimer composed of an oxygen-sensitive HIF-1α subunit and a constitutively expressed HIF-1β subunit, also known as ARNT (aryl hydrocarbon receptor nuclear translocator) [8]. While HIF-1β levels remain stable regardless of oxygen status, HIF-1α stability and transcriptional activity are tightly regulated by oxygen-dependent post-translational modifications. The induction of HIF-1α by hypoxia occurs predominantly at the protein level, as mRNA expression levels remain relatively constant irrespective of oxygen concentration [9].

Stabilized HIF-1α accumulates, translocates to the nucleus, dimerizes with HIF-1β, and binds hypoxia response elements (HREs) in the promoter and enhancer regions of target genes. This activates transcription of a broad range of genes supporting angiogenesis, erythropoiesis, glycolysis and glucose transport, pH regulation, and cell survival [10].

### 2.1. HIF-1α Regulation and Prolyl Hydroxylase Domain Proteins

**Figure 1.**
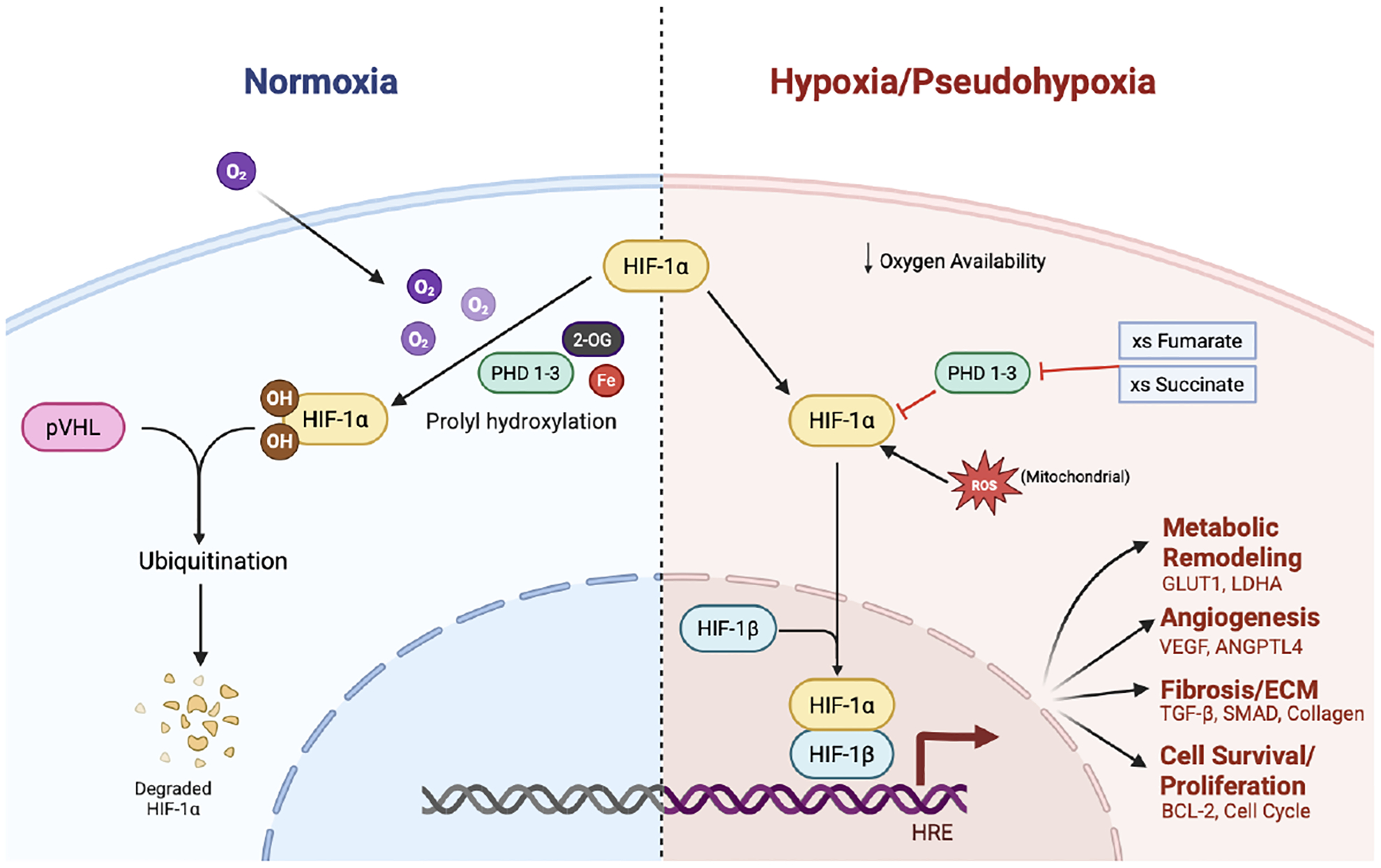
Oxygen-dependent and oxygen-independent regulation of HIF-1α signaling in normoxia and hypoxia/pseudohypoxia. Under normoxic conditions, HIF-1α is hydroxylated at proline residues by prolyl hydroxylase domain proteins (PHD1–3) in an oxygen-, iron-, and 2-oxoglutarate–dependent manner, enabling recognition by the von Hippel–Lindau tumor suppressor protein (pVHL) and subsequent ubiquitination and proteasomal degradation. In hypoxia or pseudohypoxia, reduced oxygen availability and metabolic stress inhibit PHD activity, in part through accumulation of succinate and increased mitochondrial reactive oxygen species (ROS), leading to stabilization of HIF-1α. Stabilized HIF-1α translocates to the nucleus, where it heterodimerizes with HIF-1β (ARNT) and binds hypoxia response elements (HREs) to drive transcriptional programs that promote angiogenesis, extracellular matrix remodeling and fibrosis, and cell survival and proliferation. Created with BioRender.

HIF-1α regulation depends largely on hydroxylation by prolyl hydroxylase domain (PHD) enzymes PHD1, PHD2, and PHD3, which function as intracellular oxygen sensors [11–13]. These enzymes, members of the 2-oxoglutarate-dependent dioxygenase family, require molecular oxygen, 2-oxoglutarate, and ferrous iron (Fe^2^^+^) as cofactors [14]. Under normoxic conditions, PHDs hydroxylate HIF-1α at two conserved proline residues (Pro402 and Pro564) [15,16], generating succinate and CO_2_ as byproduct [17]. Hydroxylation enables binding of the von Hippel–Lindau (pVHL) tumor suppressor protein, the substrate recognition component of an E3 ubiquitin ligase complex, leading to ubiquitination and degradation of HIF-1α by the 26S proteasome and preventing activation of hypoxia-responsive genes [18,19]. In contrast, HIF-1β-subunit remains constitutively expressed and stable independent of oxygen, ensuring availability of the dimerization partner required for transcriptional activity (Figure 1).

In hypoxia, oxygen limitation reduces PHD activity, preventing HIF-1α hydroxylation and subsequent pVHL-mediated ubiquitination [20]. As a result, HIF-1α escapes proteasomal degradation and accumulates within the cell.

Beyond oxygen availability, PHD activity and HIF-1α can be influenced by the intracellular metabolic state [21].

## 3. HIF-1α Downstream Targets in Fibroid Pathophysiology

### 3.1. Metabolic Reprogramming: Glycolytic Shift via GLUT1 and LDHA

Hypoxia-inducible factor 1α (HIF-1α) regulates a broad transcriptional program that enables uterine fibroids (leiomyomas) to adapt to chronic oxygen deprivation that is created when uterine fibroids (leiomyomas) outgrow their blood supply. Once stabilized, HIF-1α activates genes involved in multiple functional domains, including metabolic reprogramming, angiogenesis, cell survival, and ECM remodeling. Among these, metabolic reprogramming represents one of the earliest and most direct downstream consequences of HIF-1α activation, allowing uterine fibroid (leiomyoma) cells to sustain energy production under hypoxic conditions [22].

Although histologically benign, uterine fibroids (leiomyomas) exhibit a metabolic phenotype characterized by a shift towards glycolysis, a pattern commonly referred to as the Warburg effect. This shift is characterized by the increased expression of glucose transporter-1 (*GLUT1*, encoded by *SLC2A1*) and lactate dehydrogenase-A (*LDHA*), along with other glycolytic enzymes. In uterine fibroids (leiomyomas), this glycolytic reprogramming is primarily driven by HIF-1α stabilization within hypoxic tumor regions, particularly in large nodules that outgrow their vascular supply and develop poorly perfused, low-oxygen cores [23].

While the glycolytic shift is similar to metabolic reprogramming observed in malignant tumors, HIF-1α activation differs in uterine fibroids (leiomyomas) compared to malignant tumors. In malignant tumors such as endometrial cancer, HIF-1α enhance proliferation, angiogenic expansion, and metastatic progression along with oncogenic signaling pathways. However, in uterine fibroids (leiomyomas), HIF-1α supports survival in hypoxic regions, sustains metabolic homeostasis, and promotes ECM accumulation. The difference in HIF-1α activation in benign and malignant tumors shows how HIF-1α contributes to cellular proliferation without driving malignant transformation.

**Figure 2.**
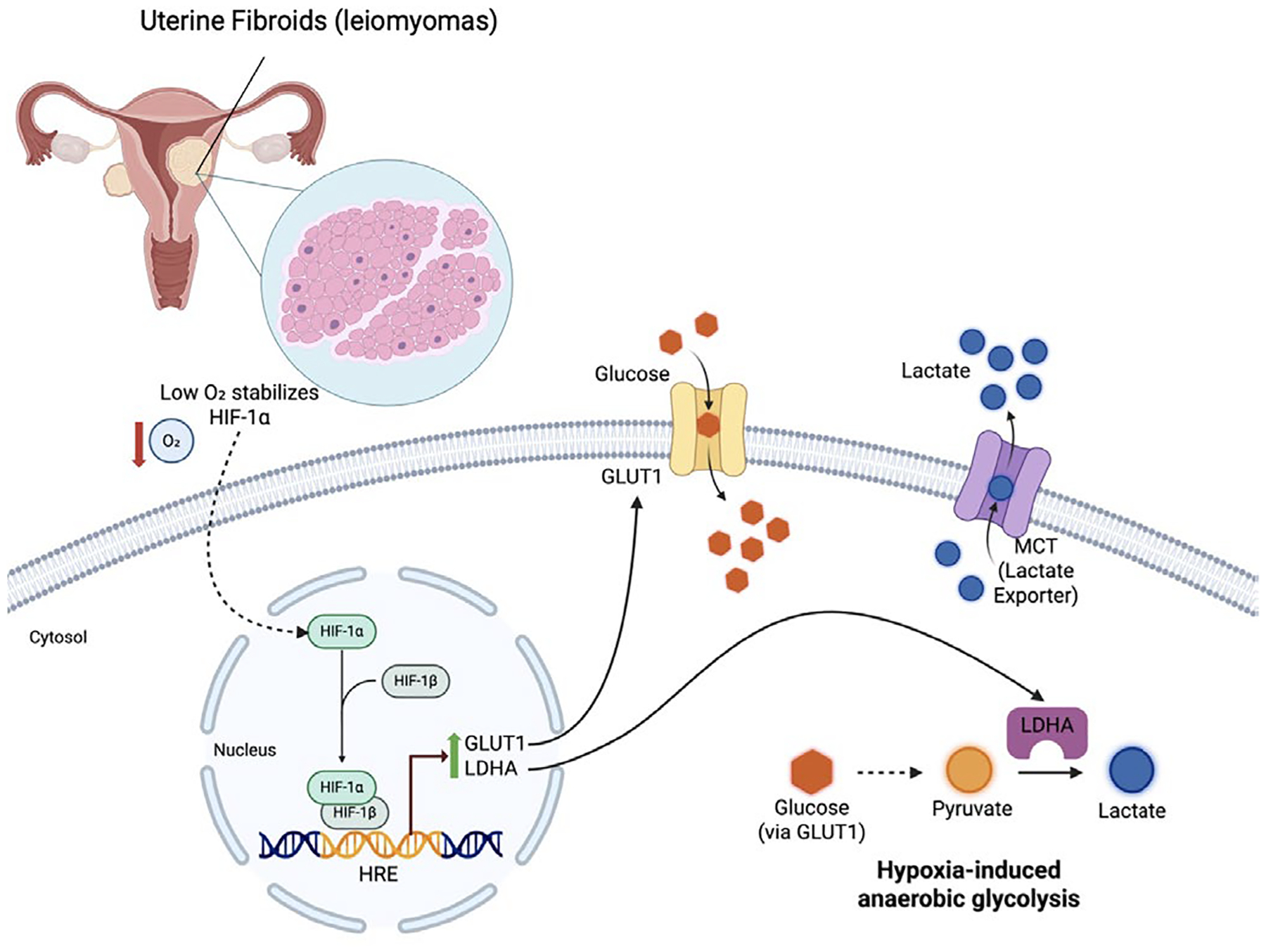
Hypoxia-induced metabolic reprogramming in uterine fibroid cells. This schematic illustrates how under hypoxic conditions, stabilization of HIF-1α promotes transcriptional upregulation of glycolytic genes, including SLC2A1 (GLUT1) and LDHA in uterine fibroids (leiomyomas). Increased GLUT1 enhances glucose uptake, while elevated LDHA drives conversion of pyruvate to lactate, which is exported via monocarboxylate transporters (MCTs). This shift toward anaerobic glycolysis supports energy production under low oxygen tension and contributes to a lactate-rich fibroid microenvironment. Created with BioRender.

At the molecular level, HIF-1α regulates glycolytic gene expression by binding HREs within target gene promoters. *SLC2A1* (GLUT1) and *LDHA* are well-established HIF-1 target genes, with hypoxia-dependent transcriptional activation demonstrated across multiple hypoxia model systems [24,25] (Figure 2).

Functionally, the upregulation of *GLUT1* enhances the high-affinity glucose uptake, ensuring substrate availability for glycolysis when oxidative phosphorylation is limited. Metabolic profiling studies indicate that uterine fibroids (leiomyomas) preferentially rely on glucose metabolism, with reduced fatty acid transport and oxidation compared with normal adjacent myometrium [26]. Increased *LDHA* expression promotes conversion of pyruvate to lactate, regenerating NAD^+^ to maintain glycolytic flux and sustaining ATP production via anaerobic glycolysis. Accumulation of lactate further contributes to extracellular acidification, which may influence local signaling and extracellular acidification within the uterine fibroid (leiomyoma) microenvironment [27].

### 3.2. Angiogenesis: VEGFA Induction, ANGPTL4, and Vascular Features

Uterine fibroids (leiomyomas) exhibit distinctive vasculature charactered by a well-vascularized peripheral capsule surrounding a relatively hypoxic, hypovascular core. This spatial heterogeneity in blood supply suggests that angiogenesis in uterine fibroids (leiomyomas) is initiated but remains structurally and functionally incomplete, resulting in immature and inefficient neovascularization [28,29]. Consistent with this pattern, fibroid-associated endometrial and myometrial tissues demonstrate disrupted vessel maturation, with fragile and poorly organized micro vessels indicative of aberrant angiogenic signaling [30]. Although uterine fibroids (leiomyomas) recruit extensive uterine arterial branching and increase overall uterine blood flow, the tumor core remains inadequately perfused, sustaining hypoxia within the fibroid tissue [31].

Uterine fibroids (leiomyomas) upregulate key hypoxia-responsive angiogenic factors, most notably vascular endothelial growth factor A (VEGF-A). VEGF-A expression is increased in uterine fibroid (leiomyoma) tissue and in the circulation of individuals with uterine fibroids (leiomyomas) compared with controls [32–34]. Circulating VEGF levels decrease following hysterectomy, supporting uterine fibroids (leiomyomas) as a major source of VEGF production [32]. Although early studies reported inconsistent associations between serum VEGF concentrations and uterine fibroid (leiomyoma) burden, more recent analyses demonstrate a significantly strong positive correlation between VEGF levels and uterine fibroid (leiomyoma) volume, indicating that VEGF expression increases with tumor size [33]. Within uterine fibroid (leiomyoma) tissue, VEGF expression is highest in poorly perfused central regions, consistent with localized hypoxia and HIF-1α activation [35]. Although VEGF-A is a known downstream target of HIF-1α in hypoxic systems, the extent to which VEGF upregulation in uterine fibroids (leiomyomas) is mediated by HIF-1α is not fully known.

In addition to VEGF, angiopoietin-like 4 (ANGPTL4) has emerged as an additional hypoxia-responsive factor with potential relevance to uterine fibroid (leiomyoma) angiogenesis. ANGPTL4 is a secreted glycoprotein with diverse functions and implications in vascular permeability and endothelial remodeling, with context-dependent effects across hypoxic and fibrotic tissues [36]. In tumor models, ANGPTL4 is induced under hypoxic conditions and can act alongside VEGF to influence angiogenic signaling and vascular integrity [37]. Evidence from fibrotic disease models suggests bidirectional interactions between ANGPTL4 and HIF-1α signaling, raising the possibility that ANGPTL4 may contribute to sustained hypoxia-driven vascular and extracellular matrix remodeling in uterine fibroids (leiomyomas) [38]. However, direct evidence supporting ANGPTL4 expression in uterine fibroids (leiomyomas) is limited.

Consistent with defective angiogenic maturation, uterine fibroids (leiomyomas) also exhibit dysregulation of vessel-stabilizing pathways. Expression of TEK (Tie2), the receptor for angiopoietin-1, has been shown to be reduced in uterine fibroid (leiomyoma) tissue relative to normal myometrium [34]. Because Ang1/Tie2 signaling is critical for vessel stabilization and maturation, its downregulation aligns with the presence of structurally immature and functionally inefficient vasculature. Together, increased expression of pro-angiogenic mediators such as VEGF, potentially augmented by ANGPTL4, alongside impaired vessel-stabilizing signals supports a model of heightened but defective angiogenesis that perpetuates hypoxia within uterine fibroid (leiomyoma) tissue [30,32].

### 3.3. Cell Cycle and Survival Pathways: Anti-apoptotic Signaling and Persistent Cell-Cycle Engagement

Within the hypoxic uterine fibroid (leiomyoma) microenvironment, cell-cycle and survival pathways are biased toward cellular persistence rather than programmed cell death [32, 39–43]. This pattern is consistent with a hypoxia-adapted state in which uterine fibroid (leiomyoma) cells remain viable and continue to expand under conditions that would typically limit smooth muscle cell survival [32].

One manifestation of this hypoxia-adapted state is elevated anti-apoptotic signaling. HIF-1α has been shown to transcriptionally regulate several genes encoding apoptosis inhibitors, including B-cell lymphoma-2 (BCL-2) and inhibitor of apoptosis-2 (IAP-2) [39]. Among these, BCL-2 is markedly overexpressed inion uterine fibroid (leiomyoma) tissue while remaining minimally detectable in normal myometrium [40]. Increased BCL-2 expression has been documented across multiple analytical platforms, including immunohistochemistry and Western blotting, indicating a robust and reproducible survival advantage in uterine fibroid (leiomyoma) cell’s [40,41]. Most of the evidence linking HIF-1α to anti-apoptotic signaling is derived from malignant tumor systems as evidence supporting HIF-1α–mediated regulation of BCL-2 in uterine fibroids (leiomyomas) is limited. Additionally, histologic studies indicate that leiomyomas rarely display features of apoptosis or necrosis, and cell loss is more commonly attributed to inanosis (fibroid cell loss attributed to chronic ischemic or nutrient deprivation) [32].

Uterine fibroids (leiomyomas) maintain sustained cell-cycle activity despite hypoxic and metabolic constraints. For example, the nuclear proliferation marker, Ki-67, is more expressed in uterine fibroid (leiomyoma) tissue (approximately ~6% of nuclei positive) than in normal myometrial tissue (~1–2%) [42]. Similarly, proliferating cell nuclear antigen (PCNA) marks cells engaged in DNA replication is readily detected in uterine fibroids (leiomyomas) but remains largely absent in normal myometrium [43]. The increased expression of Ki-67 and PCNA in uterine fibroids (leiomyomas) does not directly correlate with HIF-1α signaling as they are not specific indicators of HIF-1α pathway activation. This pattern supports a context in which HIF-1α supports cellular persistence and adaptation.

### 3.4. Estrogen and Progesterone Signaling

HIF-1α functions as an important intermediary between steroid hormone signaling and hypoxia-responsive gene regulation in hormone-responsive tissues, including the uterus. In eutopic endometrium, estrogen plays a central role in regulating angiogenesis and vascular permeability across the menstrual cycle, largely through induction of VEGF [44 – 46]. Notably, the VEGF promoter lacks a canonical estrogen response element, necessitating indirect regulatory mechanisms for estrogen-mediated VEGF induction. Experimental studies have demonstrated that 17β-estradiol (E2) promotes recruitment of HIF-1α subunits to hypoxia-responsive elements within the VEGF promoter and increases both HIF-1α mRNA and protein expression in uterine tissue [47]. This process is mediated through activation of the Phosphatidylinositol 3-Kinase/protein kinase B (PI3K/AKT) signaling pathway, linking estrogen signaling to HIF-1α stabilization, and transcriptional activity [48].

Importantly, estrogen-driven VEGF expression is functionally dependent on HIF-1α availability. In endometrial cancer models, E2 fails to induce VEGF expression under normoxic conditions unless HIF-1α is experimentally stabilized (e.g., by CoCl_2_), demonstrating that HIF-1α serves as a permissive factor required for full estrogen-mediated angiogenic signaling. Given the frequent dysregulation of the PI3K/AKT/mechanistic target of rapamycin (mTOR) pathway in uterine fibroids (leiomyomas), this estrogen–HIF-1α axis provides a mechanistic framework through which hormonal signaling may amplify hypoxia-responsive pathways in uterine fibroid (leiomyoma) tissue.

Crosstalk between estrogen signaling and HIF-1α has also been well characterized in breast cancer, where estrogen receptor-α (ERα) directly regulates HIF-1α expression via an estrogen response element within the HIF-1α gene [49]. Several genes critical to tumor growth and angiogenesis, including VEGFA, contain both estrogen and hypoxia response elements, enabling coordinated regulation by ERα and HIF-1α [49, 50]. In addition, hypoxia can activate ERα signaling in the absence of estrogen through HIF-1α-dependent mechanisms, contributing to hormone-independent growth and therapeutic resistance [51]. Although uterine fibroids (leiomyomas) are benign tumors, these findings illustrate conserved molecular mechanisms through which steroid hormones and hypoxia signaling can intersect in different hormone-responsive tissues.

Progesterone signaling also interfaces with HIF-1α, particularly in the endometrium. Progesterone is a key regulator of decidualization, a process characterized by metabolic reprogramming and increased glycolytic flux [52,53]. Progesterone administration in ovariectomized animal models induces HIF-1α expression to levels comparable to those observed in pregnancy, and progesterone-dependent activation of HIF-1α contributes to downstream hypoxia-responsive gene expression [54]. Recent evidence further suggests that progesterone-driven decidualization involves a feedback loop linking glycolysis, histone lactylation, and HIF-1α activation, reinforcing hypoxia-associated transcriptional programs [55].

In tissue where steroid hormone signaling and hypoxia coexist, HIF-1α may serve as a critical integrator that translates hormonal cues into sustained activation of hypoxia-responsive pathways, thereby contributing to uterine fibroid (leiomyoma) growth and persistence.

### 3.5. Inflammatory and Fibrotic Pathways

Chronic hypoxia within uterine fibroids (leiomyomas) activates HIF-1α–dependent signaling programs that encompass inflammatory activation and progressive fibrosis [56]. Through the control of genes involved in collagen synthesis, fibronectin deposition, and matrix metalloproteinase regulation, HIF-1α promotes extracellular matrix accumulation and perpetuates a pro-inflammatory microenvironment [1,57].

#### 3.5.1. HIF-1α–TGF-β/SMAD Signaling Drives Fibrotic ECM Accumulation

A defining feature of uterine fibroids (leiomyomas) is excessive deposition and remodeling of extracellular matrix, which constitutes a substantial proportion of tumor volume. When normalized for tissue volume, uterine fibroids (leiomyomas) contain more than twice the ECM content of normal myometrium, contributing to their characteristic firmness and altered mechanical properties [56]. This fibrotic phenotype is mechanistically linked to hypoxia-induced HIF-1α signaling through its interaction with the TGF-β/SMAD pathway.

**Figure 3.**
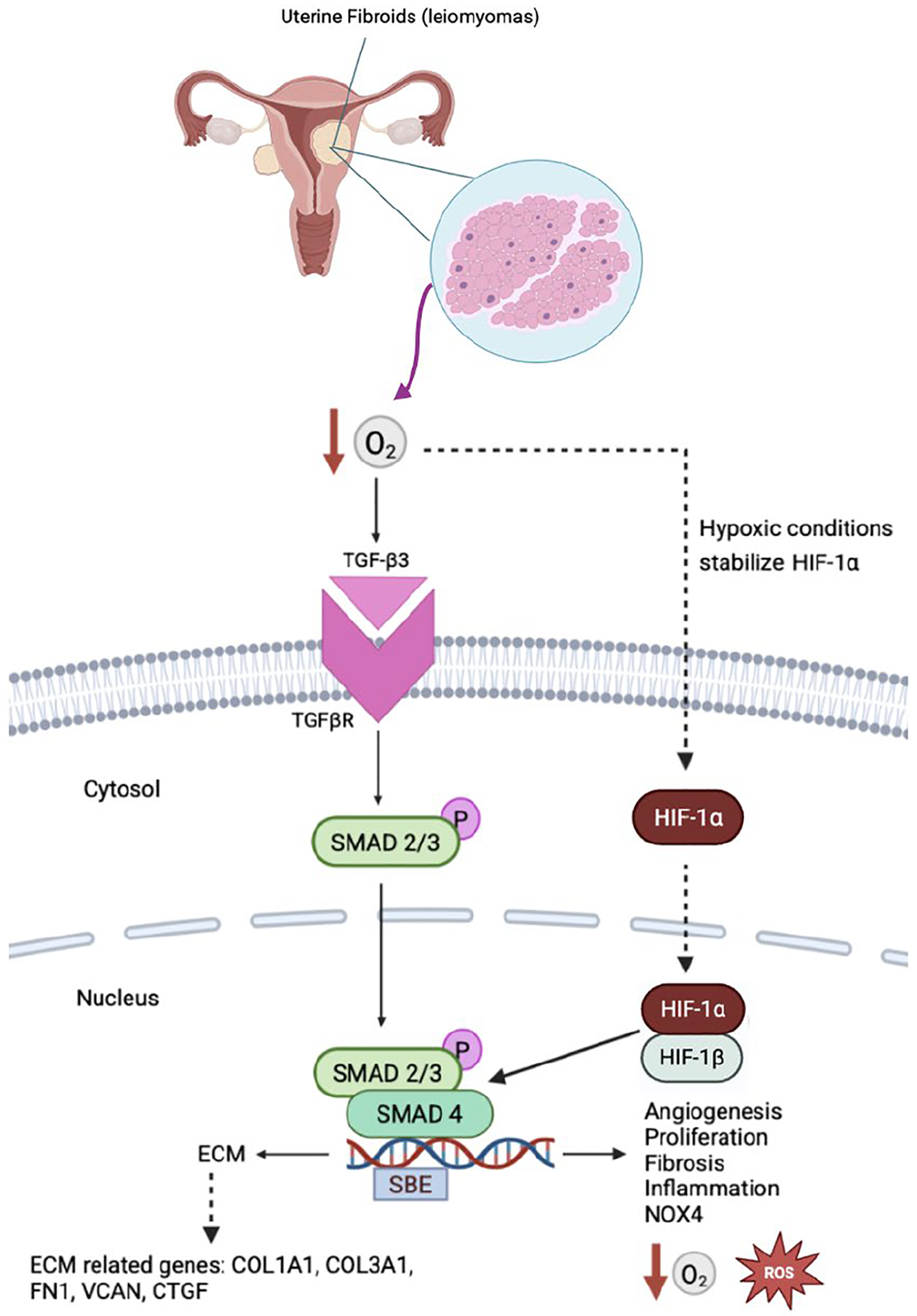
HIF-1A and TGF-β/SMAD Signaling in uterine fibroids. This schematic illustrates how under hypoxic conditions, stabilization of HIF-1α promotes activation of TGF-β3 signaling and downstream SMAD2/3–SMAD4 transcriptional complexes in uterine fibroid cells. This pathway induces expression of extracellular matrix–related genes (e.g., COL1A1, COL3A1, FN1, CTGF), contributing to excessive matrix deposition and fibrosis. Progressive ECM accumulation impairs oxygen diffusion, reinforcing local hypoxia, and sustaining HIF-1α activation. Created with BioRender.

Under hypoxic conditions, stabilized HIF-1α induces expression of TGF-β ligands, particularly TGF-β3, and promotes activation of SMAD-dependent transcriptional programs that regulate ECM gene expression [56,58, 59] (Figure 3). HIF-1α–driven SMAD2/3 phosphorylation and SMAD4 complex formation enhances transcription of key fibrotic genes, including COL1A1, COL3A1, FN1, and CTGF, while simultaneously suppressing ECM degradation through downregulation of matrix metalloproteinase activity and upregulation of tissue inhibitors of metalloproteinases (TIMPs) [58,60]. Consistent with this mechanism, transcriptomic and proteomic analyses of uterine fibroid (leiomyoma) tissue demonstrate increased expression of collagen types I and III (COL1A1, COL3A1), fibronectin (FN1), and the proteoglycan versican (VCAN) relative to matched myometrium [60–62].

HIF-1α further contributes to ECM accumulation by regulating genes that inhibit extracellular proteolysis, including SERPINE1 (PAI-1) and TIMP1, resulting in disorganized and angiogenic-prone matrix accumulation [27]. Ultrastructural analyses confirm that collagen fibrils in uterine fibroids (leiomyomas) are thicker and more irregularly arranged than in normal uterine smooth muscle, reflecting active ECM remodeling rather than simple tissue expansion [62, 63]. HIF-1α–induced SMAD activation drives the expression of ECM components such as collagen, fibronectin, and tenascin-C, resulting in their excessive deposition and accumulation within the uterine fibroid (leiomyoma) tissue. As ECM accumulates, increased tissue stiffness impairs oxygen diffusion and disrupts vascular architecture, reinforcing localized hypoxia, and sustaining HIF-1α activation [64–67].

#### 3.5.2. Interplay Between HIF-1α and NF-κB–Mediated Inflammation

In parallel with fibrotic signaling, HIF-1α engages in reciprocal crosstalk with inflammatory pathways mediated by nuclear factor-κB (NF-κB). NF-κB is activated in uterine fibroid (leiomyoma) tissue by pro-inflammatory cytokines, growth factors, and oxidative stress. Once activated, NF-κB induces expression of inflammatory cytokines (TNF-α, IL-1β, IL-6), chemokines (MCP-1, RANTES), and adhesion molecules (ICAM-1, VCAM-1), promoting immune cell recruitment and angiogenesis within the uterine fibroid (leiomyoma) microenvironment [7].

Pro-inflammatory cytokine signaling can stabilize HIF-1α and enhance its transcriptional activity even when oxygen levels are not severely reduced, linking inflammatory signaling directly to hypoxia-responsive gene expression [68]. In uterine fibroid (leiomyoma) cells, HIF-1α activation promotes expression of glycolytic enzymes and glucose transporters, increasing lactate production and contributing to local acidification and reduced oxygen availability [69]. Together, these interactions help maintain a tissue environment in which inflammation, hypoxia, and fibrotic remodeling persist.

Comparable interactions between HIF-1α, TGF-β, and NF-κB have been described in other fibrotic diseases, including hepatic fibrosis, where NF-κB–dependent macrophage signaling supports myofibroblast survival and extracellular matrix deposition [70–73]. In uterine fibroids (leiomyomas), dysregulation of these interconnected pathways shifts normally reparative responses toward chronic inflammation and fibrosis, supporting slow but sustained tumor growth [74].

### 3.6. mTOR/AMPK-Mediated Modulation of HIF-1α Activity

Regulation of HIF-1α signaling and transcriptional activity in uterine fibroids (leiomyomas) extends beyond oxygen-dependent stabilization to include nutrient- and energy-sensing pathways.

**Figure 4.**
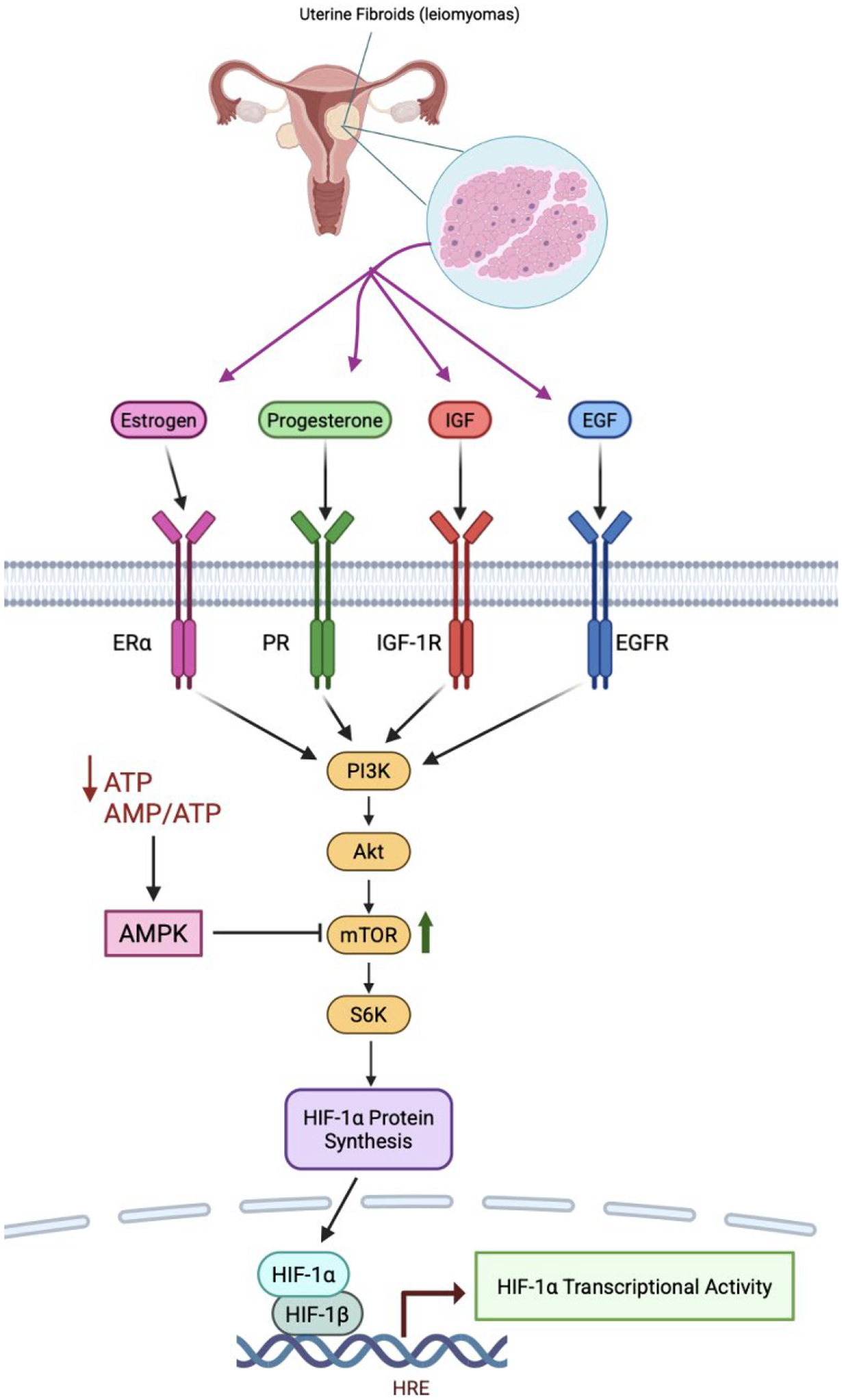
Metabolic regulation of HIF-1α signaling via mTOR and AMPK in uterine fibroids. This schematic illustrates how nutrient- and energy-sensing pathways regulate HIF-1α abundance independently of oxygen availability in uterine fibroid cells. Estrogen activates estrogen receptor alpha (Erα) and progesterone activates the progesterone receptor (PR). Insulin-like growth factor-1 (IGF-1) binds to the insulin-like growth factor 1 receptor (IGF-1R) and epidermal growth factor (EGF) activates the epidermal growth factor receptor (EGFR). These receptors activate PI3K/AKT/mTORC1, enhancing HIF-1α protein synthesis, while AMPK opposes this process under conditions of energetic stress. In fibroids, sustained mTOR activation favor persistent HIF-1α signaling and downstream transcriptional programs. Created with BioRender.

mTOR complex 1 (mTORC1) is a central regulator of protein synthesis and cellular growth that directly influences HIF-1α at the translational level (Figure 4). Activation of the PI3K/AKT/mTOR pathway by growth factors such as insulin-like growth factor (IGF) and epidermal growth factor (EGF) increases HIF-1α protein synthesis under normoxic conditions [75].

Uterine fibroids (leiomyomas) exhibit hyperactivation of the PI3K/AKT/mTOR axis, consistent with strong hormonal and growth factor inputs [76]. Estrogen, progesterone, IGF, and EGF converge on PI3K/AKT signaling, resulting in persistent mTORC1 activation [75]. This signaling promotes cell survival, angiogenesis, and extracellular matrix accumulation – processes that substantially overlap with HIF-1α downstream transcriptional programs. Preclinical models support a causal role for mTOR activation in uterine fibroid (leiomyoma) development: in the Tsc2-haploinsufficient Eker rat, constitutive mTOR signaling drives uterine fibroid (leiomyoma) formation, while rapamycin analogs significantly reduce tumor incidence, multiplicity, and size [77, 78]. Evidence from in vitro models further supports this interaction. In ELT-3 uterine fibroid (leiomyoma) cells, pharmacologic inhibition of mTOR with rapamycin or activation of AMP-activated protein kinase (AMPK) with metformin suppresses VEGF expression through an mTORC1–HIF-1α–dependent mechanism [79]. Collectively, these findings identify mTOR as both a driver of uterine fibroid (leiomyoma) pathogenesis and an upstream amplifier of HIF-1α signaling.

In contrast to mTOR, AMPK functions as an energy-sensing brake that is activated by increased AMP/ATP ratios during metabolic stress [80]. AMPK activation is triggered by cellular energy depletion, glucose deprivation, oxidative stress, and upstream kinase signaling through liver kinase B1 (LKB1) in response to the calcium flux [80]. Once activated, AMPK suppresses mTORC1 activity, reduces HIF-1α translation, and limits anabolic biosynthetic pathways under low-energy conditions [81]. In uterine fibroids (leiomyomas), this counter-regulatory restraint may be attenuated by chronic hormonal and growth factor signaling that sustains mTOR activation [82]. Nonetheless, AMPK remains pharmacologically targetable. Metformin, a classical AMPK activator, has been shown to reduce hypoxia-driven VEGF expression in uterine fibroid (leiomyoma) cells, consistent with suppression of mTORC1–HIF-1α–linked outputs [79].

## 4. HIF-1α and Redox Imbalance

In uterine fibroids (leiomyomas), dysregulated redox homeostasis represents an additional distinct mechanism by which HIF-1α activity is sustained independent of local oxygen tension [83]. Reactive oxygen species (ROS), generated through altered cellular metabolism and inflammatory signaling, interferes with canonical HIF-1α degradation pathways. Specifically, ROS impair prolyl hydroxylase–mediated hydroxylation of HIF-1α, limiting its recognition by the pVHL complex and promoting protein stabilization and transcriptional activation [84]. Through this mechanism, oxidative stress reinforces HIF-1α signaling within the uterine fibroid (leiomyoma) and supports downstream programs associated with fibrosis. In hypoxia-adapted tumor contexts, stabilized HIF-1α promotes glycolytic gene expression and suppresses mitochondrial oxidative metabolism, including through paracrine effects on stromal and cancer-associated fibroblast populations [85]. In uterine fibroids (leiomyomas), these metabolic shifts may further contribute to ROS generation, creating a reinforcing interaction between redox imbalance and hypoxia-responsive signaling.

Beyond direct effects on HIF-1α stability, ROS activate multiple upstream signaling cascades, including MAPK and PI3K/AKT pathways, which enhance HIF-1α expression and transcriptional activity [85]. Oxidative stress also influences extracellular matrix dynamics by modulating enzymes involved in collagen synthesis and degradation, thereby contributing to ECM accumulation and fibrotic remodeling characteristics of uterine fibroid (leiomyoma) tissue [86].

### 4.1. Impaired Antioxidant Defenses in Fibroid Tissue

In uterine fibroids (leiomyomas), attenuation of endogenous antioxidant systems favors continued ROS accumulation, creating conditions that support sustained HIF-1α activity. Reduced expression of mitochondrial superoxide dismutase 2 (SOD2) and catalase has been reported in uterine fibroid (leiomyoma) tissue relative to matched myometrium [87]. Loss of these enzymes limits detoxification of superoxide radicals and hydrogen peroxide, shifting intracellular redox balance toward persistent ROS accumulation. This environment favors continued stabilization of HIF-1α.

Deficient antioxidant defenses can also significantly alter downstream signaling behavior. Elevated ROS activate redox-sensitive transcription factors, including NF-κB and AP-1, amplifying inflammatory signaling pathways that intersect with HIF-1α–dependent transcriptional programs. Additional antioxidant systems, such as glutathione peroxidases and peroxiredoxins, may also be compromised in uterine fibroids (leiomyomas), further reducing the capacity to buffer oxidative stress and prolonging redox driven signaling [88].

Excessive ROS accumulation leads to oxidative damage of lipids, proteins, and DNA. This damage contributes to cellular dysfunction, inflammation, and tissue injury, all of which promote fibrogenesis and uterine fibroid (leiomyoma) progression. This pathological redox state contrasts with the tightly regulated role of ROS in normal ovarian physiology, where transient ROS signaling supports follicular development, ovulation, and luteal function [89]. While moderate ROS levels participate in physiological transcriptional regulation and steroidogenesis [90], sustained oxidative stress is associated with reproductive pathology, including infertility, polycystic ovary syndrome, and ovarian malignancy [90,91]. In uterine fibroids (leiomyomas), chronic disruption of redox homeostasis reinforces inflammation, fibrosis, and persistent HIF-1α signaling.

## 5. Fumarate Hydratase Deficiency and Hereditary Leiomyomatosis and Renal Cell Carcinoma Syndrome

Tricarboxylic acid (TCA) cycle metabolites can modulate oxygen-sensing pathways through their effects on α-ketoglutarate–dependent dioxygenases. Fumarate and succinate have emerged as important regulators of hypoxia signaling because their accumulation interferes with the enzymatic reactions required for HIF hydroxylation. Loss of fumarate hydratase (FH), the mitochondrial enzyme that catalyzes the conversion of fumarate to malate, leads to intracellular fumarate accumulation [92,93].

FH deficiency is most prominently observed in Hereditary Leiomyomatosis and Renal Cell Carcinoma (HLRCC) syndrome, an autosomal dominant disorder caused by germline pathogenic variants in FH [94]. Individuals with HLRCC develop multiple cutaneous uterine fibroids (leiomyomas) and early-onset uterine fibroids (leiomyomas) that are often numerous and symptomatic, while remaining at risk for an aggressive form of renal cell carcinoma [94–96]. HLRCC tumor development follows a tumor suppressor model. A germline FH mutation is followed by somatic loss of the remaining functional allele within affected tissues, resulting in complete loss of FH activity [94].

Importantly, FH deficiency in benign uterine fibroids (leiomyomas) is not restricted to hereditary disease. Pathologic and genomic studies demonstrate that a subset of these tumors exhibit FH loss through somatic biallelic inactivation. This indicates that fumarate-driven tumor biology can arise in sporadic uterine fibroids (leiomyomas) independent of germline HLRCC [97,98]. FH-deficient uterine fibroids (leiomyomas) therefore represent a distinct molecular subgroup within the broader spectrum of uterine fibroids (leiomyomas) [97].

Accumulated fumarate, together with the related TCA metabolite succinate, can inhibit α-ketoglutarate–dependent enzymes including prolyl hydroxylases [92]. This interference disrupts canonical HIF degradation pathways. As a result, HIF transcription factors can remain stabilized even under normoxic conditions. This state is commonly described as metabolic pseudohypoxia [92,93]. Through this mechanism, mitochondrial metabolic dysfunction becomes linked to activation of hypoxia-responsive transcriptional programs that regulate angiogenesis, metabolic adaptation, and extracellular matrix remodeling (Figure 1).

## 6. Therapeutic Targeting of HIF-1α in Fibroids

Rather than functioning as a single downstream effector, we have described HIF-1α integration into hypoxia, steroid hormone signaling, growth factor input, metabolic rewiring, and inflammatory cues. This network architecture creates multiple potential therapeutic entry points through which HIF-1α–dependent uterine fibroid (leiomyoma) growth can be attenuated.

### 6.1. Direct HIF-1α Inhibition in Fibroids

Direct inhibition of HIF-1α suppresses key pathogenic processes in uterine fibroid (leiomyoma) models. In primary human uterine fibroids(leiomyoma) cells cultured under hypoxic conditions, echinomycin and PX-478 reduce proliferation and increase apoptosis at pharmacologically relevant concentrations [99]. KC7F2 similarly lowers HIF-1α protein levels and reduces hypoxia-induced secretion of VEGF-A, adrenomedullin, and endothelin-1 [100,101]. These effects are accompanied by reduced expression of proliferation markers such as PCNA.

Efficacy has also been demonstrated in vivo using a hormone-supported NOD/SCID kidney capsule xenograft model. Treatment with direct HIF-1α inhibitors, including echinomycin and PX-478, significantly reduced Ki-67 indices and decreased graft size. Immunohistochemical analysis confirmed reduced intralesional HIF-1α following treatment [99]. Comparable antiproliferative effects were observed when therapy was initiated shortly after graft implantation and when administered to established tumors, indicating that HIF-1α inhibition remains effective across distinct stages of uterine fibroid (leiomyoma) growth under physiologic hormone support.

Even though antiproliferative effects are detectable in myometrial cells in vitro, clinical translation is limited by pharmacokinetic constraints, which include short half-life, systemic exposure, and off-target toxicity. Early-phase clinical oncology trials of PX-478 report dose-limiting toxicities such as fatigue and gastrointestinal effects, highlight the need for improved delivery strategies that target uterine fibroids (leiomyomas)while preserving normal uterine function in adjacent tissue (Table 1).

### 6.2. Hormone-directed Therapies that Down-regulate HIF-linked Outputs

Hormonal therapies offer an indirect means of limiting HIF-1α–dependent signaling within the uterine fibroid (leiomyoma) microenvironment and may complement direct HIF-1α inhibitions. Progesterone receptor modulation reduces expression of angiogenic and vasoactive factors that lie downstream of HIF-1α transcriptional activity, thereby dampening hypoxia-amplified vascular signaling in uterine fibroid (leiomyoma) cells [102].

In patient-derived xenografts, progesterone antagonism with mifepristone arrests or reverses tumor growth, while mTOR inhibition produces similar reductions in graft size under identical steroid support conditions [103]. Although these agents do not directly target the HIF complex, they diminish hormonal signaling that supports continued expression of HIF-1α–dependent angiogenic and metabolic genes, positioning hormone modulation as a rational therapy adjunct. This therapeutic logic mirrors approaches used in hormone-responsive malignancies, where receptor-directed interventions indirectly suppress hypoxia-associated transcriptional programs and improve disease control [104,105].

Clinically, selective progesterone receptor modulators (SPRMs) such as ulipristal acetate have shown to reduce uterine fibroid (leiomyoma) volume and control bleeding, even though the concern hepatoxicity have restricted long-term use [106]. Recently, relugolix combination therapy, an oral gonadotropin-releasing hormone (GnRH) antagonist, has shown significant reduction in menstrual bleeding and uterine fibroid (leiomyomas size in phase III trials (Table 1) [107]. These two therapies have been FDA-approved and suppress HIF-1α activity by reducing the growth signals that sustain angiogenesis.

### 6.3. Upstream Pathway Inhibitors that Converge on HIF-1α Translation and Activity

An alternative strategy to suppress HIF-1α translation in uterine fibroids (leiomyomas)is to target pathways that regulate HIF-1α synthesis and stability upstream of the transcriptional factor itself. As described above, persistent activation of the PI3K/AKT/mTOR pathway enhances translation of HIF-1α mRNA and sustains conditions consistent with metabolic pseudohypoxia [83].

Pharmacologic disruption of this axis attenuates HIF-linked signaling. In uterine fibroid (leiomyoma) cell models, metformin suppresses VEGF expression through AMPK-mediated inhibition of mTORC1, reducing HIF-1α–dependent transcriptional activity [108]. In vivo, mTOR inhibition decreases uterine fibroid (leiomyoma) burden in both xenograft systems and the Tsc2-haploinsufficient Eker rat, a genetically driven model of uterine fibroid (leiomyoma) formation [77, 84]. These effects are accompanied by reduced proliferation and downstream pathway activity, consistent with diminished HIF-1α signaling.

Findings from oncology studies demonstrate the mechanistic relevance of this pathway. In solid tumors, mTOR blockade limits HIF-1α translation and shortens protein half-life, leading to reduced angiogenic signaling and altered metabolic gene expression [102,104]. Because uterine fibroids (leiomyomas) rely on the same upstream growth and metabolic inputs, inhibition of mTOR represents a rational means of indirectly suppressing HIF-1α activity in hormonally responsive uterine tissue. Lastly, mTOR inhibition in these settings can also improve the effect of other targeted drugs, including anti-angiogenic therapies [79].

In clinical trials, sirolimus and everolimus (mTOR inhibitors) have demonstrated antiproliferative effects in preclinical uterine fibroids (leiomyomas) models, but the clinical data is limited. Their systemic immunosuppressive effects present challenges for long term use (Table 1) [109]. The translation to uterine fibroid (leiomyoma) therapy will require improved delivery systems to treat uterine fibroids ((leiomyoma) while limiting off-target effects.

## 7. Conclusion and Future Directions

Hypoxia, steroid hormone signaling, growth factor input, inflammation, and metabolic remodeling all reinforce HIF-1α activity. The therapeutic relevance of HIF-1α in uterine fibroids (leiomyoma) lies in its position as a convergence point for a larger signaling network rather than in a single downstream effector pathway. Effective intervention therefore requires strategies that disrupt this network without compromising normal myometrial function.

Future therapeutic development should prioritize a rational combination of approaches that exploit these complementary mechanisms. Experience from oncology studies indicates that HIF-targeted therapies are most effective when paired with agents that limit compensatory signaling and prolong pathway suppression. In uterine fibroids (leiomyoma), such combinations may allow lower dosing, reduce off-target effects, and preserve normal uterine physiology. Despite this, key challenges remain including achieving sufficient drug delivery exposure to uterine fibroids (leiomyoma)while minimizing systemic toxicity.

Long-term safety must be established for interventions that alter hypoxia-responsive signaling in reproductive tissues. Additionally, further research into patient-specific factors, including genetic predispositions and racial differences, is crucial to understanding their influence on HIF-1α expression and activity within uterine fibroids (leiomyoma) tissue. This understanding will facilitate the development of personalized treatment strategies, enhance therapeutic efficacy, and address current limitations in uterine-sparing fibroid management.

## Figures and Tables

**Table 1.** Summary of Therapeutic Strategies Targeting HIF-1α Signaling in Fibroids.

Strategy	Mechanism	Clinical Status	Limitations
Direct HIF-1α inhibitors (PX-478 and echinomycin) [99]	Block HIF transcriptional activity	Preclinical/ Phase I	Toxicity, system exposure, pharmacokinetics
SPRMs (ulipristal acetate) [106]	Progesterone receptor modulation	Approved, Restricted Use	Hepatotoxicity
GnRH antagonist (relugolix) [107]	Suppress estrogen/progesterone signaling	FDA approved	Hormonal side effects
Metformin [108]	AMPK inactivation, mTOR inhibition	Investigational	Limited RCT evidence
mTOR inhibitors (sirolimus, everolimus) [109]	Block HIF-1α translation	Not FDA approved	Systemic toxicity, immunosuppression

## Abbreviations

The following abbreviations are used in this manuscript:

HIF-1α: Hypoxia-inducible factor-1α
ECM: Extracellular matrix
ARNT: Aryl hydrocarbon receptor nuclear translocator
HRE: Hypoxia response elements
PHD: Prolyl hydroxylase domain
pVHL: von Hippel–Lindau
TCA: Tricarboxylic acid
ROS: Reactive oxygen species
GLUT1: Glucose transporter-1
LDHA: Lactate dehydrogenase-A
MCT: Monocarboxylate transporters
VEGF-A: Vascular endothelial growth factor A
ANGPTL4: Angiopoietin-like 4
BCL-2: B-cell lymphoma-2
IAP-2: Inhibitor of apoptosis-2
PCNA: Proliferating cell nuclear antigen
PI3K/AKT: Phosphatidylinositol 3-Kinase/protein kinase B
ERα: Estrogen receptor-α
TIMP: Tissue inhibitors of metalloproteinase
NF-κB: Nuclear factor-κB
IGF: Insulin-like growth factor
EGF: Epidermal growth factor
SOD2: Superoxide dismutase 2
FH: Fumarate hydratase
mTORC1: mTOR complex 1
AMPK: AMP-activated protein kinase
LKB1: Liver kinase B1
HRLCC: Hereditary Leiomyomatosis and Renal Cell Carcinoma syndrome
